# The Lang Youth Medical Program: a 6-year blueprint for advancing educational attainment and achievement

**DOI:** 10.3389/fmed.2025.1734475

**Published:** 2026-01-26

**Authors:** Madeleine Ripple, Aysha Tabassum, Ronald R Sanchez, Isabelle Elton, J. C. Alejaldre, Mara Minguez

**Affiliations:** 1 Division of Community and Population Health, NewYork-Presbyterian Hospital, New York, NY, United States; 2 Department of Pediatrics, Columbia University Irving Medical Center, New York, NY, United States

**Keywords:** career exploration, educational attainment, enrichment programming, health sciences education, pipeline program, youth development

## Abstract

**Introduction:**

Medical pipeline programs are one upstream approach to supporting student educational outcomes, a well-documented social determinant of health, through out-of-school time (OST) programming. The Lang Youth Medical Program (LYMP) is a 6-year, hospital-based model designed to immerse students (“Scholars”) in a health science and clinical curriculum, fostering interest in health careers and providing educational support for youth in Northern Manhattan. LYMP stands out among other medical pipeline programs for its longitudinal model that targets students in middle school to enable comprehensive academic and career support. This paper aims to describe the structure, implementation, and outcomes of LYMP as a replicable model for improving educational outcomes among underrepresented youth.

**Program overview:**

LYMP draws upon an urban hospital's existing resources and community partnerships to immerse Scholars in academic and clinical exploration. Across 6 years, Scholars receive a body-systems-based curriculum, clinical skills lessons, hospital tours, and annual summer internships, alongside rigorous support for high school and college applications, to prepare them for post-secondary pursuits. Simultaneously, Scholars' families receive parent workshops and connections to wraparound and counseling services as an additional dimension of support.

**Outcomes:**

With a 92.6% retention rate across 16 graduating cohorts, approximately 93% of LYMP alumni pursue undergraduate degrees and one in five have pursued graduate degrees. Among alumni who have graduated college, 26% (*n* = 30) have returned to work full-time at the hosting hospital. Regardless of their professional or academic pathway, recent LYMP alumni scored 27% higher on standardized tests compared to their peers and report feeling well-equipped with professional and academic skills instrumental in their success.

**Discussion:**

LYMP offers a scalable and replicable blueprint for healthcare institutions seeking to invest in educational equity and workforce development. Its modular curriculum, upstream intervention strategy, and integration within a hospital setting demonstrate how longitudinal, community-rooted programs can effectively support youth in achieving academic success, career readiness, and health literacy. Additionally, LYMP's novel approach has evolved to include socioemotional support and community resources to address the social determinants of health, providing Scholars with a holistic exposure to healthcare while continually meeting its mission to improve educational outcomes among Northern Manhattan youth.

## 1 Introduction

Education is a well-established social determinant of health, influencing both individual and communal health. Individuals who do not graduate high school engage in riskier health behaviors, such as smoking cigarettes or are less physically active, have poorer self-rated health, and increased mortality (1–3). Research demonstrates that increased educational attainment is associated with increased health literacy, enabling individuals to follow medical instructions, navigate healthcare systems, and make positive health decisions more easily (2). Individuals with a high school diploma and bachelor's degree are also more likely to secure a job that offers higher pay and employer-provided health insurance, factors that contribute to greater economic stability and improved healthcare access (1).

The education-health relationship is observable across New York City (NYC) neighborhoods. As of 2022, 22.6% of residents in NYC's Washington Heights/Inwood (WHI) neighborhood in Northern Manhattan have not completed high school, compared to 15.8% of NYC residents overall (4). Only 39.7% of individuals obtain at least a bachelor's degree, slightly less than the citywide rate of 42.5%, despite nearly two-thirds of WHI's most recent high school graduates enrolling in post-secondary education (4). Only 40 and 48.5% of WHI third graders are proficient in English Language Arts and math, respectively, falling short of the citywide rates of 48 and 55% (4). Approximately 15% of youth aged 16–24 are disconnected, meaning that they are not involved in education or employment (4).

Healthwise, data demonstrates a significant chronic disease burden among WHI residents. Among both children and adults, approximately one in four experience obesity (4). Approximately 21.4% of WHI adults have a depression diagnosis, the sixth highest rate in NYC, and 34.6% of WHI adults have high cholesterol (4). WHI adults also engage in poorer health behaviors, including binge drinking (19.2%) and eating no fruits or vegetables (19%). Overall, 68% of WHI adults self-rate their health as “excellent,” “very good,” or “good,” compared to 78% citywide, placing WHI as the fourth lowest neighborhood in self-rated health in NYC. Although these data reflect adult outcomes, they highlight community health patterns that often begin in adolescence, underscoring the need for early, sustained youth interventions like our program to foster healthier behaviors and prevent the continuation of these disparities into adulthood.

Out-of-school time (OST) programs are a longstanding approach to providing students with supplemental academic support. OST programs occur outside of regular school hours, either during the academic year, summer months, or school breaks, and must include an academic component, such as supervised homework time or dedicated lessons on specific subjects. Research shows that OST programs improve grade point averages, test scores, and school attendance for both middle and high school students and hold potential to assist youth with overcoming learning barriers and developing social skills (5, 6). Furthermore, participation in Science Technology Engineering and Math (STEM) focused OST programming may support long-term motivation to pursue STEM-related education and careers after high school (7, 8). While literature regarding non-STEM OST programming's effects on post-secondary education and career endeavors is limited, program evaluations examining STEM OST participation and post-secondary plans suggest that early exposure to and engagement with specific topics through academic rigor, collaboration, and real-world connections fosters student curiosity and self-efficacy that may carry through to related advanced studies and careers (7, 8).

Youth-centered medical pipeline programs are one category of OST programming that provide students with academic exploration, academic support, and career exploration. Existing programs focus on healthcare careers, college readiness, STEM preparation, or conducting community-based healthcare projects through short-term summer or academic-year initiatives, often requiring a 1-year commitment during students' high school or undergraduate years (9–11). Among programs that primarily serve high school students, ample evaluations provide evidence that medical pipeline programs improve college matriculation rates, Standardized Admissions Test (SAT) scores, and self-efficacy among participants (12–14). However, limited studies look at career choices and long-term impacts job exploration elements, such as mentorship and professionalism. In NYC, the State Pre-College Enrichment Program at the Columbia University Vagelos College of Physicians and Surgeons provides academic support and health topic exposure to students between 7th and 12th grade, offering 1–6 years of support, depending on the student. Similarly, the Weill Cornell Medicine's Health Profession Recruitment/Exposure Program combines weekly health career exploration and college preparation but targets only tenth and eleventh-grade students.

In WHI, NewYork-Presbyterian Hospital (NYPH) established the Lang Youth Medical Program (LYMP) at its Columbia University Irving Medical Center campus in 2003 to strengthen academic-community partnerships and support neighborhood youth. LYMP is a one-entry point, 6-year program for students interested in medicine and health careers. At no cost to their families, LYMP recruits a cohort of 6th grade students to progress through a rigorous “mini-medical school,” public health, and professional preparation curriculum, immersing students in the health sciences and healthcare spaces. Since its inception, LYMP has provided academic, professional, and personal development to over 250 youth, including 76 active participants and 179 graduates, 75 of whom have pursued health-related studies and careers.

LYMP provides a distinct longitudinal approach that targets students early in their academic trajectory. Where many youth-centered medical pipeline programs emphasize short-tern exposure or single-summer engagements through health education and mentorship, limited literature documents the range and depth of guidance and support provided to participants and families to aid student development and academic achievement. Most medical pipeline programs also exist within medical schools or universities and miss the opportunity to connect participating youth with vital resources often available only within hospital settings, like behavioral health support, internships, and job shadowing, that further support students in their academic journeys. LYMP's approach is novel not just for its longevity but for its commitment to leveraging hospital resources to support both students and families throughout program participation. Lastly, limited literature evaluating youth-centered medical pipeline programs document participants' long-term career outcomes in healthcare. LYMP incorporates a structured alumni component that extends support and advising beyond program completion, supporting continuity and long-term educational persistence and enabling close data tracking of alumni outcomes.

LYMP's 6-year pipeline model offers a strategic approach to improving long-term health outcomes by addressing educational attainment, a critical social determinant of health. Through a structured combination of academic enrichment, experiential learning, sustained mentorship, and family support, LYMP equips youth with the knowledge, skills, and exposure necessary to reach key educational milestones, pursue higher education, and career advancement. In strengthening academic and career pathways, LYMP mitigates the early emergence of health disparities by reinforcing protective factors that are known to influence long-term wellbeing, such as educational success, reduced psychosocial stressors, and improved access to future opportunities. LYMP aims to:

- Increase youth awareness of healthcare professions and health topics through a comprehensive health science curriculum and mentorship.
- Support youth through higher education and the health career pathway through longitudinal, individualized academic counseling, and test preparation.
- Equip youth with professional skills for sustained success through professional development workshops and internships.

This paper aims to describe the structure, implementation, and outcomes of the LYMP as a replicable model for healthcare institutions seeking to invest in community health, educational outcomes, and workforce development among underrepresented minority youth.

## 2 Key programmatic elements

### 2.1 Participant recruitment

Each spring, LYMP recruits a cohort of 15–17 current 6th grade students in Northern Manhattan and the Bronx to start in the fall of their 7th grade year. Working closely with school administrators and educators, LYMP relies on school presentations and program open houses to engage prospective Scholars. LYMP's four-pronged screening process includes an electronic application, a group interview, an individual student interview, and a parent interview to ensure that all accepted Scholars and families are the best fit for the program. Accepted Scholars come from numerous backgrounds, experiences, and schools but most commonly embody the following characteristics:

1. Academic excellence, including strong school performance and attendance
2. Excitement toward STEM and the human body
3. Curiosity in exploring medicine and healthcare

### 2.2 Health science and public health curriculum

LYMP's 6-year model employs a comprehensive, systems-based approach, where each semester contains a six-lesson module on one body system, exposing Scholars to a range of health disciplines and how they can expect to learn in a graduate health program. Each system module follows a similar lesson cadence and covers the following topics:

1. Anatomy and Physiology
2. Pathology
3. Diagnosis and Treatment Methods
4. Personal Health
5. Community Health
6. Global Health

LYMP's curriculum leverages a student-centered learning approach, seeking to foster a collaborative, project-based learning environment that promotes academic engagement, autonomy, and self-efficacy and equips Scholars with the academic and research skills necessary for fostering long-term interest in health (15). Weekly lessons interweave historical, socioemotional, scientific, and public health concepts with NYC-based examples to promote active inquiry about the root causes of health issues and critical thinking about real-life relevance of concepts.

LYMP curates all activities to foster solution-oriented, community-conscious thinking and clinical reasoning as though Scholars were practicing healthcare professionals. Scholars actively participate in their learning through hands-on activities, such as dissections and modeling body systems using household objects, and service-learning opportunities with partnering community-based organizations. Scholars engage in peer-to-peer instruction by presenting their research findings to each other and have case-based discussions at the end of each lesson to reinforce concepts. Each academic year culminates in a year-end symposium where Scholars collaborate in small groups to research and create a presentation on a chosen topic—which is later presented to LYMP staff, hospital staff and faculty, physician, residents, medical students, families, and community members.

### 2.3 Health career exploration, immersion, and mentorship

LYMP emphasizes experiential learning within the clinical setting and from other health care professionals. NYPH's affiliation with Columbia University and Cornell University enables LYMP Scholars to regularly interact with healthcare spaces and professionals in varying career stages to bolster their career development and inform their understanding of health career possibilities. For example, LYMP recruits its weekly instructors from its robust alumni network and NYPH's affiliated graduate health programs, and medical residents conduct monthly clinical skills lessons and clinical space tours through NYPH.

Each summer after completing 9th grade, Scholars participate in 6-week, full-time internships at NYPH, receiving mentorship from healthcare professionals and hands-on exposure in various clinical settings. By their graduation, Scholars complete 720 h across three internships in hospital spaces. Many Scholars also maintain contact with internship preceptors post-program graduation, providing them with mentors across health professions as they proceed with their post-secondary and professional endeavors.

In addition to hands-on experiences, LYMP emphasizes health career immersion by celebrating milestones designed to mirror that of medical students. Scholars receive short and long white coats in 8th and 9th grade, signifying their progression through LYMP's rigorous curriculum. LYMP also celebrates an annual match day, modeled after the national residency match day, where Scholars learn their internship placements for that summer. Such events expose Scholars to key steps in a medical doctor's professional journey.

### 2.4 Academic preparation

Acknowledging that post-secondary outcomes are closely related to high school rigor and success (16), LYMP offers individualized assistance with high school and college applications, standardized test preparation, and scholarship opportunities. Because LYMP starts in the fall of 7th grade, program offerings aim to educate and prepare middle school Scholars about NYC's competitive high school application process and admission into NYC's highly ranked high schools. Scholars engage in high school fairs and lessons curated for the middle-to-high school transition to ensure they are well-supported throughout their high school careers.

Scholars are expected to maintain a minimum grade of 80% in all coursework throughout their 6 years and receive individualized support from LYMP when falling below that benchmark. Scholars also receive regular standardized test preparation for the Specialized High School Admissions Test, Pre-Standardized Admissions Test, and the Standardized Admissions Test (SAT). Moreover, Scholars receive one-on-one support for college applications, concentrating on writing personal statements, formulating college lists, and identifying scholarship opportunities.

### 2.5 Family support

LYMP recognizes the importance of familial support in student success and strives to collaborate with families to ensure that all members are well-supported during Scholars' participation. Throughout the academic year, families receive workshops about the high school and college application processes, academic readiness, and socio-emotional development to ensure that they, too, are well-supported during Scholars' progression toward post-secondary and career endeavors. All family-facing program materials and activities are also translated into Spanish to ensure accessibility for all members of the predominantly Spanish-speaking community that LYMP serves.

## 3 Outcomes

LYMP collects outcome data through self-reported surveys and feedback from current participants and program alumni. LYMP's alumni coordinator performs outreach to all program alumni biannually to update education and employment information. Alumni information is also collected through informal touchpoints with individuals throughout the year. All participants sign media and information releases upon matriculation into the program for quality improvement purposes.

### 3.1 College admissions

Scholars graduating between 2021 and 2024 had an average SAT score of 1,116, 16% higher than the NYC average and 27% higher than the NYC average for Black and Latinx students (17). Five Scholars have received academic accolades, including one Fulbright Scholar, two Posse Scholars, and two Hispanic Scholarship Fund Scholars. Of the 53 alumni currently enrolled at an undergraduate institution, approximately 70% (*n* = 37) are on track to graduate debt-free.

### 3.2 Alumni achievement

As of 2024, LYMP's graduation and retention rate is 92.7%, boasting 179 alumni across 16 graduating classes. Among these alumni, 92.8% (*n* = 166) have confirmed attendance at a 4-year institution, and 1.2% (*n* = 2) have enlisted in the military.

Health-related career pathways remain popular among LYMP alumni. A recent evaluation of alumni experiences finds that the LYMP widened their perspectives on possible health careers (15). As of 2024, approximately 42% of alumni have graduated with or are actively pursuing a premedical or health science major, including two in a combined Bachelor of Science-Doctor of Medicine program. Among 114 alumni who have completed their undergraduate education, 31.5% (*n* = 36) have pursued graduate degrees, including 22.8% (*n* = 26) who have pursued graduate health degrees and four who have pursued multiple graduate degrees (Table 1). Thirty alumni have returned to NYPH as full-time hospital employees in clinical or non-clinical roles.

**Table 1:** Graduate degrees in-progress and/or completed by LYMP alumni as of 2024.

**Graduate degree type**	**Number of alumni**
Master of Social Work (MSW) or Licensed Mental Health Counseling (LMHC)	6
Master of Science (MS)	6
Doctor of Medicine (MD)	4
Master of Science in Nursing (MSN)	4
Master of Business Administration (MBA)	3
Master of Public Administration (MPA)	3
Master of Public Health (MPH)	3
Doctor of Philosophy (PhD)	2
Doctor of Physical Therapy (DPT)	2
Master of Arts (MA)	2
Master of Fine Arts (MFA)	2
Doctor of Osteopathic Medicine (DO)	1
Doctor of Occupational Therapy (OTD)	1
Master of Science in Occupational Therapy (MSOT)	1

LYMP alumni are positioned for personal, professional, and academic success even if they choose not to pursue a health-related career. In cases where a Scholar does not choose to pursue a career in healthcare, alumni cite LYMP's role in helping them identify whether or not healthcare was the best pathway for them through its robust career exposure activities (18). Alumni recognize LYMP's structure and curriculum as a supporter of their resilience, time management, health literacy, and leadership skills (18). Academically, alumni report developing skills that well-prepared them for their undergraduate coursework. More tangibly, LYMP provided alumni with ample support for job applications, such as resume writing and interviewing skills, and upheld high professional standards, which alumni report eased their transition into the workforce upon completing their education (18).

## 4 Discussion—Lessons learned and constraints

The Lang Youth Medical Program provides an upstream intervention to higher education attainment in a Northern Manhattan neighborhood through a 6-year, clinically immersive model that emphasizes early career exploration and rigorous academic support. Affirmed by its growing number of alumni pursuing post-secondary studies and health careers requiring advanced degrees, LYMP has made significant strides toward achieving this goal and accomplishes an additional, if not equally important, objective: promoting a lifelong commitment to personal and communal wellbeing. LYMP Scholars graduate with health literacy and communication skills that empower them to make better health decisions as adults and to act as the next generation's health advocates, regardless of their profession. Such a model serves as a blueprint for holistic program development and implementation that is cognizant of the needs and leverages the resources of the community it serves.

LYMP largely attributes its success, particularly its high retention and completion rate, to its intensive recruitment model and early incorporation in Scholars' academic journeys. Scholars are selected based on academic performance and attendance, as well as demonstrated motivation to pursue advanced education and careers. These factors provide a strong foundation for Scholars entering the program to meet the rigor of health science education while most benefiting from the mentorship and experiential learning opportunities provided by the program. To build on this, LYMP recruits from neighborhoods where Scholars live, and involves parents early in the interview process to ensure family engagement. Additionally, by launching programming in the 7th grade, LYMP ingrains program participation in Scholars' routines before the incorporation of additional extracurricular commitments in high school. Family and Scholar engagement are integral to program completion, and LYMP's early contact with participants coupled with its multi-step recruitment model ensures Scholars are set up for success throughout their tenure in the program.

LYMP's sustainability and success are further supported by a braided funding model that leverages multiple institutional and philanthropic resources. Instead of being dependent on a sole and specific funding source, the program strives to embed programmatic stagging, the expertise of clinically trained hospital staff and university faculty, physical space, educational materials, and family-facing resources and interventions across various departments throughout the hospital system. This approach improves program stability and flexibility when funding sources have changed or are time limited.

An additional benefit is that a braided funding model allows for interdepartmental collaboration and combined institutional ownership, integrating the program into the hospital's tapestry rather than viewing it as an expendable program model. Acknowledging that students' wellbeing, families, and immediate communities play a critical role in their educational and professional outcomes, leveraging hospital resources through such a model is integral to LYMP fulfilling its mission. For hospital systems interested in implementing or sustaining like-minded longitudinal tough development and career pathway models, braided funding models may provide a realistic strategy to lessen the burden and dependence on time-specific resources while upholding program continuity and impact.

LYMP's two-decade lifespan has also experienced challenges affecting its sustainability and operations that are worth noting for institutions hoping to replicate similar models. Over time, LYMP has evolved to address obstacles encompassing alumni support, hospital compliance, curriculum development, and community partnerships to ensure that it best supports participants in all dimensions to best fulfill the program mission.

First, supporting an individual through the health career pipeline—from middle school through undergraduate education—is, at minimum, a 10-year commitment. It is important to ensure that students enroll in undergraduate education to pursue a health career, but it is equally, if not more, important to ensure that they remain supported throughout their undergraduate studies to reach that goal. NYPH developed LYMP to support a student for the first 6 years of this timeline, but LYMP has further developed its alumni support to ensure that it is meeting its mission of improving student academic achievement and health career attainment. This includes implementing a dedicated alumni coordinator to address individual alumni needs and scholarships to provide financial support.

Second, LYMP's hands-on approach requires all Scholars to onboard as NYPH volunteers to access clinical areas and participate in summer internships. Despite close guidance by LYMP and Volunteer Services staff during the onboarding process, LYMP loses at least one incoming Scholar each year due to challenges with the onboarding and medical compliance processes. To address this hospital compliance challenge alongside others, LYMP has maintained strong collaborations with NYPH departments to introduce additional layers of communication, support, and public health education to ensure that families understand the requirements, can make informed decisions, and are equipped with the appropriate resources to support Scholar compliance. Institutions seeking to replicate LYMP's approach must understand hospital compliance protocols to proactively create a system to educate and guide incoming families through the required onboarding and medical steps and build-in any renewal activities for continuing participants.

Third, LYMP's current curriculum is modular, meaning each lesson is extractable by body system or lesson topic and designed to emphasize real-world, hands-on learning. A modular curriculum has allowed LYMP to take an interdisciplinary approach in teaching Scholars about medicine and health topics and fostering critical skills while keeping its content age-appropriate and rigorous for each grade level. Implementing a modular curriculum also provides a degree of scalability where programs can tailor lessons to fit their objectives and resources. Where this approach has its strengths, combining a modular curriculum format with LYMP's student-centered approach requires routine updates to maintain relevance to Scholars' daily lives and reflect evolving technology, community issues, healthcare systems, and scientific knowledge. Requiring regular curriculum updates also requires having program staff that are knowledgeable and adept at curriculum development to sustain rigor and Scholar engagement.

Lastly, LYMP's longitudinal model ensures that Scholars receive early and ample exploration opportunities and targeted support at all steps in their academic, personal, and professional development. Strong partnerships with hospital and academic departments and community organizations are a cornerstone to this approach. Where LYMP has longstanding relationships with numerous NYPH departments, community organizations, and area schools, the nature of such partnerships may vary over time as personnel, leadership, and priorities change. As such, LYMP's relationships with internal and external partners has evolved alongside LYMP's programming to ensure a mutually beneficial, trusting relationship among all partners.

Despite its demonstrated success, LYMP requires further evaluation to assess its outcomes, impact, and program components. In alignment with previously published medical pipeline program evaluations, LYMP demonstrates efficacy in improving college matriculation rates, SAT scores, and promoting participants to study health sciences. However, LYMP data currently lacks information regarding changes in Scholars' self-efficacy and general attitudes toward healthcare careers. However, such outcomes can be inferred from LYMP's robust alumni career outcome data. To fill gaps in its measurable outcomes, LYMP is currently undergoing an evaluation on the impacts of its multi-year internship model on Scholars' educational and career trajectory, including their interest in pursuing a career in healthcare. Future research is also necessary to examine selection bias' influence on program outcomes using a control group of participants.

## Data Availability

The original contributions presented in the study are included in the article/supplementary material, further inquiries can be directed to the corresponding author.
